# Simultaneous beating-heart mitral valve replacement and aortic repair following Bentall procedure via double right mini-thoracotomies: a case report

**DOI:** 10.1186/s44215-024-00168-0

**Published:** 2024-09-20

**Authors:** Toshimasa Tanaka, Takeshi Kinoshita, Daisuke Endo, Minoru Tabata

**Affiliations:** Department of Cardiovascular Surgery, Juntendo University Graduate School of Medicine, 2-11-6 Hongo, Bunkyo-Ku, Tokyo 113-0033 Japan

**Keywords:** Double right mini-thoracotomy approach,, Beating-heart surgery, Redo cardiac surgery, Minimally invasive cardiac surgery

## Abstract

**Background:**

Redo mitral valve surgery by re-sternotomy approach has challenges such as bleeding and organ injury due to adhesion dissection, sternal bone infection, and poor field of view of mitral valve. On the other hand, redo mitral valve surgery via a right mini-thoracotomy approach appears to address these challenges. We successfully performed a double right mini-thoracotomies approach for mitral valve replacement and pseudoaneurysm repair under the beating-heart condition. Herein, we report the effectiveness and the safety of this technique and detailed procedure.

Case presentation.

The patient is a 71-year-old man with a history of Bentall procedure using a mechanical valve at another hospital 30 years ago. He developed acute heart failure due to severe mitral valve regurgitation. After medication, he was referred to our department for the purpose of surgery for mitral valve regurgitation. Preoperative transesophageal echocardiography showed extensive degenerative change of the both leaflets and chordae tendineae rupture at the P3 segment. Both left atrium and ventricle dilated, and left ventricle contractility reduced. Aortic mechanical valve had no problem. In addition, preoperative contrast enhanced computed tomography revealed a pseudoaneurysm at the distal anastomotic site of Bentall procedure. We performed mitral valve replacement by mechanical valve and repair of distal anastomotic cite under beating heart condition, utilizing a double right mini-thoracotomies approach for mitral valve and ascending aortic pseudoaneurysm respectively. The postoperative course was uneventful, the patient was discharged without complications.

**Conclusion:**

The right mini-thoracotomies approach efficiently accessed mitral valve and ascending aorta in reoperations, reducing the adhesion dissection risks and ensuring clear exposure. Moreover, concomitant use of beating-heart technique minimized adhesion dissection for aortic cross-clamp, preserved cardiac function.

## Background

Redo mitral valve surgery via a right mini-thoracotomy (RMT) approach minimizes adhesion dissection and provides optimal exposure of the mitral valve, especially following aortic valve replacement (AVR) [1]. There have been few reports of combined reoperations including mitral valve surgery and aortic procedure by the RMT approach. By utilizing a double RMT approach, we successfully performed beating-heart mitral valve replacement (MVR) and repair of ascending aortic pseudoaneurysm following Bentall procedure. We report the detailed procedure and its advantages.

## Case presentation

**Fig. 1:**
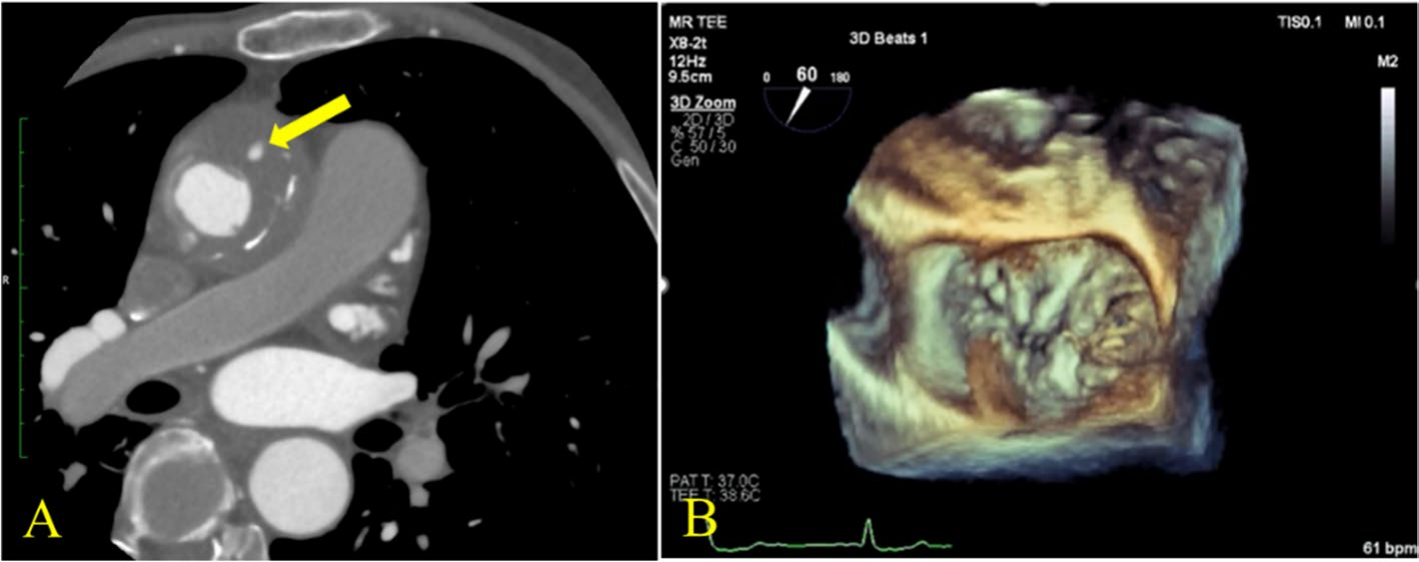
**A** Preoperative contrast-enhanced CT showing contrast leakage at the distal anastomosis following Bentall procedure. **B** Preoperative transesophageal echocardiography demonstrating extensive degenerative change of both leaflets and P3 prolapse due to chordal rupture

The patient is a 71-year-old man with a history of Bentall procedure using a mechanical valve at another hospital 30 years ago for aortic valve regurgitation (AR) and aortic root dilation associated with Marfan syndrome. He developed severe mitral valve regurgitation (MR) 6 months ago, requiring hospitalization. Once his heart failure stabilized, he was referred to our department for surgical intervention to severe MR. He was receiving anticoagulation therapy with warfarin for chronic atrial fibrillation. Preoperative contrast-enhanced CT revealed a hematoma encircling the graft of the ascending aorta and a contrast extravasation from distal anastomosis; indicating a pseudoaneurysm at the distal anastomotic site (Fig. 1a). Preoperative transthoracic echocardiography showed that the end-diastolic and end-systolic diameters of the left ventricle were 61 mm and 42 mm, respectively, and the left ventricular ejection fraction was 59%. The aortic mechanical valve was functioning well. Moreover, preoperative transesophageal echocardiography showed severe MR due to extensive degenerative change of the both leaflets and chordae tendineae rupture at the P3 segment. The annulus was severely dilated, and all leaflet segments were redundant (Fig. 1b). The anteroposterior diameter of the mitral annulus was 44.3 mm; the commissural diameter was 53.3 mm, the lengths of A2, P1, P2, and P3 were 31.7 mm, 15.2 mm, 21.3 mm, and 18.9 mm, respectively.

**Fig. 2:**
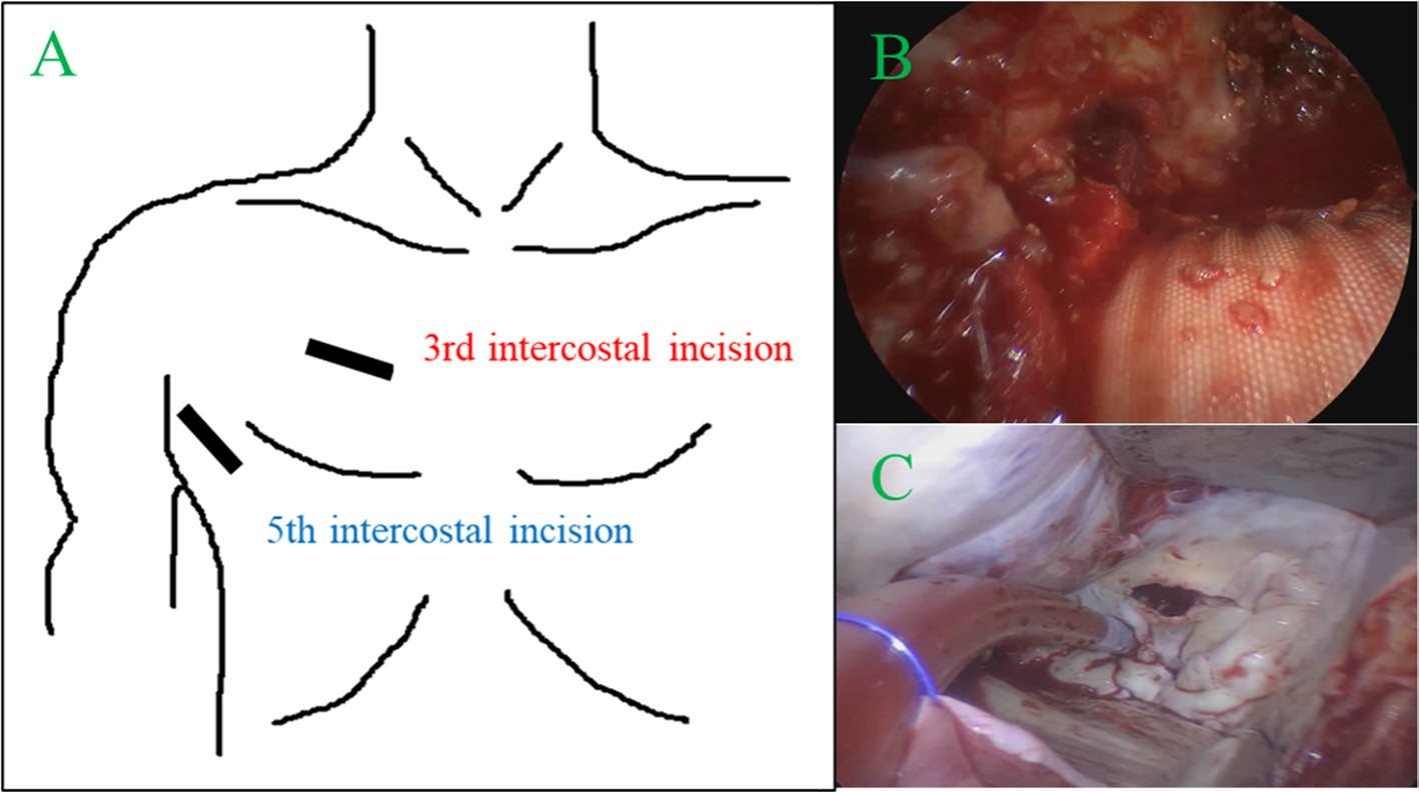
**A** Double right mini-thoracotomies approach. **B** The repair site of the aortic anastomosis by direct vision approach via the third intercostal mini-thoracotomy. **C** The mitral valve exposure by endoscopic approach via the fifth intercostal mini-thoracotomy

We opted for mechanical mitral valve replacement (MVR) and an anastomotic site repair using a beating-heart RMT approach. We made two mini-thoracotomies: a 5-cm incision in the right anterior axillary line at the fifth intercostal space for MVR and a 4-cm incision in the right anterior chest at the third intercostal space for the pseudoaneurysm repair (Fig. 2a). We added a 5-mm endoscopic port through the fifth intercostal space and placed a carbon dioxide insufflation and left ventricular vent tube through the sixth intercostal space. Cardiopulmonary bypass was established with the cannulation at right femoral artery and vein, and right internal jugular vein. The procedures were performed under beating-heart conditions as body temperature naturally decreased to 34.6 °C without active cooling. Under direct vision, the aortic bleeding site was identified. The size of pseudoaneurysm was the same as that of preoperative CT, and there were no findings of infection. Moreover, the surrounding tissue was intact. So, we repaired with pledgeted 3–0 polypropylene horizontal mattress sutures (Fig. 2b). Subsequently, a venting cannula was placed in the ascending aorta. With the Trendelenburg position, we opened the left atrium, ensuring continuous venting of the ascending aorta, left ventricle, and left atrium to prevent air embolism and to maintain a bloodless field. With a totally endoscopic approach, the MV was excellently exposed and subsequently replaced with a 29-mm SJM mechanical valve using a chordal preserving technique and pledgeted non-everting mattress sutures (Fig. 2c). The operating time was 240 min, and the cardiopulmonary bypass time was 164 min. He was discharged on the 8th postoperative day without complications.

## Discussion and conclusions

This reoperation case was unique due to the concurrent presence of two distinct conditions, severe MR and ascending aortic pseudoaneurysm following Bentall procedure. Re-sternotomy approach carries high risks of bleeding, tissue injury, and sternal infection. Therefore, we believed that the RMT approach would reduce the risks of re-sternotomy and provide clear mitral valve visualization, despite the post-AVR context. In this case, we needed to repair the anterior aspect of the ascending aorta. Because the ideal access for MVR was not suitable for a pseudoaneurysm repair, we made two strategic incisions in advance. A double RMT approach minimizes adhesion dissection and enhances the effectiveness and safety of the procedure. This approach could offer significant advantages even for primary combined surgeries.

Ascending aortic pseudoaneurysms can occur at the site of graft anastomosis, aortotomy, cannulation, or proximal anastomosis of coronary artery bypass grafting [2]. The most common etiology is infection, with other possible etiologies related to the surgical technique, including thread deterioration, as well as the presence of connective tissue disorder that cause fragility of the aortic wall, such as Marfan syndrome [2]. In this case, the inflammatory marker was normal at the time of surgery, and there was no active graft infection. Additionally, the size of ascending aorta distal to graft was not enlarged, indicating that the enlarged diameter of the native aorta was not the cause of pseudoaneurysm. It is suspected that previous infection and fragility of the native aortic tissue may have led to anastomotic failure and the pseudoaneurysm.

Preoperative contrast-enhanced CT showed that the pseudoaneurysm was localized in the anterior wall of the distal anastomosis, just under the right third intercostal space. Based on the intraoperative findings, we considered that repairing the pseudoaneurysm would be feasible via a right mini-thoracotomy. However, if this approach proved inadequate, we planned to cool down the body temperature with placing a left ventricular vent, extend the right anterior thoracotomy, and repair the ascending aorta under hypothermic circulatory arrest. In this case, we opted MVR using mechanical valve rather than mitral valve repair. According to the results of preoperative transthoracic echocardiography, left ventricle enlarged and left ventricular ejection fraction was likely overestimated due to severe MR. Furthermore, the patient had chronic atrial fibrillation and was already taking warfarin. In addition to these elements, the mitral valve was highly degenerated; therefore, we considered the high risk of recurrent MR after mitral valve repair [3].

Another crucial element of our strategy was the implementation of beating-heart surgery. In redo minimally invasive cardiac surgery, tissue dissection between the ascending aorta and pulmonary artery or within the transverse sinus is required for aortic cross-clamping. However, after Bentall procedure, it is not difficult to assume that the tissues surrounding aorta and pulmonary artery are tightly adhered. This method minimized the adhesion dissection required for aortic cross-clamping and avoided potential aortic injury from endo-balloon clamping. Although the ventricular fibrillation surgery is another option, it reduces oxygen delivery to the left ventricle, potentially inducing subendocardial ischemia [4]. Consequently, moderate or deeper hypothermia is essential. In contrast, beating-heart surgery preserves subendocardial circulation and eliminates the need for moderate or deeper hypothermia [4]. Therefore, beating-heart surgery was suitable for this case with reduced left heart function.

Moreover, we effectively prevented air embolism by continuously venting the ascending aorta and left ventricle and filling the surgical field with carbon dioxide. These preventative measures helped to avoid systemic air embolism.

On the other hand, it is important to note that the beating-heart minimally invasive mitral valve surgery is not suitable in cases with significant AR, even if mild, due to poor visualization. However, this approach is appropriate for cases where AR is trivial or less.

In conclusion, the beating-heart double RMT approach proved to be highly effective for concurrent MVR and anastomotic repair following Bentall procedure. This efficient novel strategy holds promise for combined reoperations involving the mitral valve and aorta.

## Data Availability

Data sharing is not applicable to this article as no datasets were generated or analyzed during the current study.

## Abbreviations

RMT: Right mini-thoracotomy
AVR: Arterial valve replacement
MVR: Mitral valve replacement
AR: Aortic valve regurgitation
MR: Mitral valve regurgitation
